# Methodological Challenges for Epidemiologic Studies of Deprescribing at the End of Life

**DOI:** 10.1007/s40471-021-00264-7

**Published:** 2021-04-23

**Authors:** Jennifer Tjia, Jennifer L. Lund, Deborah S. Mack, Attah Mbrah, Yiyang Yuan, Qiaoxi Chen, Seun Osundolire, Cara L. McDermott

**Affiliations:** 1 Department of Population and Quantitative Health Sciences, University of Massachusetts Medical School, 368 Plantation Street, AS6-2065, Worcester, MA 01605, USA; 2 Department of Epidemiology, UNC Gillings School of Global Public Health, UNC Chapel Hill, Chapel Hill, NC, USA; 3 Division of Pulmonary, Critical Care and Sleep Medicine, University of Washington, Seattle, WA, USA

**Keywords:** Deprescribing, Medication appropriateness, End of life, Methods, Outcome measurement, Methodological

## Abstract

**Purpose of Review:**

To describe approaches to measuring deprescribing and associated outcomes in studies of patients approaching end of life (EOL).

**Recent Findings:**

We reviewed studies published through 2020 that evaluated deprescribing in patients with limited life expectancy and approaching EOL. Deprescribing includes reducing the number of medications, decreasing medication dose(s), and eliminating potentially inappropriate medications. Tools such as STOPPFrail, OncPal, and the Unnecessary Drug Use Measure can facilitate deprescribing. Outcome measures vary and selection of measures should align with the operationalized deprescribing definition used by study investigators.

**Summary:**

EOL deprescribing considerations include medication appropriateness in the context of patient goals for care, expected benefit from medication given life expectancy, and heightened potential for medication-related harm as death nears. Additional data are needed on how EOL deprescribing impacts patient quality of life, caregiver burden, and out-of-pocket medication-related costs to patients and caregivers. Investigators should design deprescribing studies with this information in mind.

## Introduction

Deprescribing is a term referring to the process of reducing inappropriate polypharmacy by discontinuing, withdrawing, or tapering medications under the direction of a healthcare provider [1]. The goal of deprescribing is to minimize potential medication risks and to achieve better patient outcomes [2].

The end of life is an important time for deprescribing, particularly for older adults, because polypharmacy is common [3–5] in the face of progressively declining organ function that increases older adults’ risk of drug-related harm [6–8]. Many medications at end of life are of questionable benefit [9–11], yet most medications continue until death, while the number of end-of-life (EOL) symptom management drugs increases [5]. This changing combination of medications contributes to stress for patients and caregivers [12,13].

Studying deprescribing at end of life is important but methodologically challenging. One issue is operationalizing the definition of deprescribing. Another issue is the selection of appropriate outcome measures. Designing valid clinical trials and observational studies must balance numerous factors, including validity, utility, psychometric properties of measures, and relevance to stakeholders.

To address these challenges, we conducted this narrative review of published peer-reviewed literature to describe approaches to the measurement of deprescribing and related outcomes for studies of patients at end of life. The intention is to highlight, for a range of domains of interest to deprescribing stakeholders, what strategies have been used, whether they can be used in studies relying upon primary or secondary data collection approaches, and what may be desirable but needs further research to bring into the deprescribing toolbox. This paper will serve as a resource for investigators conducting studies in this growing area of research.

## Methods

For this narrative review, the authors conducted a literature search in November to December 2020 of MEDLINE (PubMed), Embase, Web of Science, Google Scholar, and PsychLit for relevant empirical studies and review articles published up to December 31, 2020. Search terms included deprescribing, discontinuation, end-of-life, palliative, caregiver, outcomes, methods, and polypharmacy. We included retrospective and prospective studies conducted in humans and published in English, including observational, interventional, and cross-sectional study designs. A manual search of reference lists of included articles was performed to ensure the inclusion of relevant articles and resources. Heterogeneity across studies precluded pooling data, and herein we present results in narrative format. We first describe several methods for defining deprescribing and then organize relevant outcome measures aggregated by patient and family caregivers and into the following domains: medication-related outcomes, patient-reported outcomes, clinical outcomes, safety outcomes, and cost.

## Results

### Deprescribing

Deprescribing includes the following: (1) drug discontinuation, (2) deintensification (i.e., gradual dose reduction without discontinuation), or (3) switching from inappropriate to appropriate drugs. Operationalizing the deprescribing definition depends on the study design, objectives, and conceptual definition of deprescribing. Observational studies may describe the prevalence of discontinuation and deintensification, while interventional studies may focus on lowering overall polypharmacy, reducing the number of inappropriate drugs, or stopping a particular class of drugs. Each of these aspects of deprescribing raise different measurement challenges.

Determination of whether discontinuation, deinten sification, or both should be included in a study can depend, in part, on the clinical recommendations for deprescribing which are specific to each drug class. Referencing drug-specific deprescribing guidelines can ensure that the more generalized definition applied (e.g., tapering vs abrupt discontinuation) represents the true prevalence of deprescribing in a population. Drug-specific recommendations for tapering vs discontinuation are emerging [14, 15·]. Examples of commonly accepted recommendations for select medications appear in the Supplemental Table. For best practice, investigators may include sensitivity analyses to compare results with various deprescribing definitions.

Below we highlight approaches to operationalizing discontinuation and gradual dose reduction in observational studies.

### Discontinuation/Gap Periods in Drug Exposure

One challenge to operationalizing drug discontinuation is the dynamic nature of drug prescribing, the unavailability of medical records for many studies, and the lack of explicit notation stating an unequivocal intention to stop medications when medical records are available. One general approach frequently used to define discontinuation relies on identifying gap periods in medication use. Gap periods have included 7 days [16], 14 days [17], and 30 days [18] between drug fills in published end-of-life studies based on pharmacy dispensing and claims. Non-end-of-life studies have also used 60 days [19] or 90 days [20]. Specific study examples appear in Table 1. In addition, gap periods can be determined by drug-specific pharmacokinetic properties (e.g., drug discontinuation defined as a washout period of five times the drugs half-life, representing complete excretion of the drug and biologically corresponding to an expected physiological response) [21].

### Deintensification and Gradual Dose Reduction

Deprescribing may involve dose reduction without discontinuation. Defining and operationalizing gradual dose reduction is challenging because administrative claims data often lack detailed data on drug doses. Thus, operationalizing this aspect of the deprescribing approach remains largely underexplored. One important recent review [22··] highlights specific challenges, while describing innovative composite dosage intensity measures and quantified deintensification within three therapeutic classes: central nervous system agents, antihypertensives, and antidiabetic agents. Of note, each drug class required a different composite measure. For example, antihypertensive drug intensity uses defined daily dose (DDD) or maximum recommended daily dosage in the denominator. This approach has been successfully applied using hospice medication administration records in one Veterans Administration-based study [16] (Table 1).

After defining deprescribing, identifying appropriate outcome measures from the patient and caregiver perspectives is critical.

### Patient Outcomes

Outcomes of interest to patients can be considered in terms of medication burden, medication-related clinical outcomes, quality of life effects, and economic impact.

### Medication-Related Outcomes

#### Number of Medications

Number of medications and prevalence of polypharmacy are common deprescribing outcome measures. Polypharmacy is defined as the concurrent use of multiple medications surpassing a given threshold, typically 5 (“polypharmacy”) and 10 (“excessive polypharmacy”). In practice, the operationa lization of polypharmacy is inconsistent across studies in terms of the type of data available, length of measurement time, and medications counted towards “polypharmacy.”

When prescription information is available in patients’ complete health records, a pharmacotherapy review can provide an accurate “point prevalence” (i.e., prevalence at a particular point of time) of patients’ medication use by day [23] or during the data collection period [24, 25·, 26··, 27··]. When such patient-level data is not available, linked registry databases [28··, 29·] or administrative datasets such as the Minimum Data Set [30] have been used to determine the “period prevalence” (i.e., prevalence over an interval of time) of polypharmacy. Measurement time for polypharmacy may range from “day” [23], “month” [28··, 29·], to “year” [25·].

### Medication Regimen Complexity

Since medications vary in dosing frequency, a cumulative, composite measure of overall dose administration offers informative insights into drug burden beyond simple drug counts. For this purpose, the Medication Regimen Complexity Index (MRCI) [31, 32] characterizes complexity of a patient’s medication regimen. MRCI weights dosage form, frequency, and administration instructions. Higher MRCI score is associated with unplanned hospitalizations [33], increased mortality [34], and nonadherence [34]. It has been used in studies of residential aged care facilities [35], palliative care interventions [36], and in a pilot trial of deprescribing in home hospice patients [Clinicaltrials.gov NCT03972163]. Implementing use in studies can be labor intensive, and efforts to automate MRCI calculation [37] have not yet eliminated the need for manual data entry.

### Medication Appropriateness

Types of medications used are also important to consider. Determining medication appropriateness is consistent with the goal of improving the quality and safety of a patient’s treatment plan. Medication appropriateness can be broadly defined using either implicit or explicit criteria. Implicit criteria are judgement-based indicators that focus primarily on the patient rather than drugs or diseases [38]. Explicit criteria are clearly defined lists of potentially inappropriate medications (PIMs). Implicit approaches tend to require time-consuming review by knowledgeable clinicians [39], thus increasing demand on investigator resources when used.

### Implicit Measures

For end-of-life studies, the Unnecessary Drug Use Measure defines unnecessary medications based on a simplification of the Medication Appropriateness Index. This definition includes medications that lack an indication, lack effectiveness, or are used for prolonged duration. One uncontrolled, retrospective study in geriatric palliative care simplified regimens by reducing unnecessary drug use by 65% (from 1.7 to 0.6 per patient) [40·] (Table 2).

### Explicit Measures

The Screening Tool of Older Persons’ Prescriptions in Frail adults (STOPPFrail) was a major advance in the field. STOPPFrail, developed in 2017, is an explicit list of 27 medications that may be inappropriate in frail older adults aged 65 years or older with limited life expectancy [41]. It is based on literature review and Delphi consensus process. A study comparing the STOPPFrail to a structured geriatricianled deprescribing process (gold standard) among 100 clinical cases reported a positive predictive value of 89.3% [42]. This approach is clinically applicable and efficient, taking 3 min to apply when reviewing regimens of 10 drugs on average, and has high interrater reliability [42]. In a randomized controlled trial, it successfully reduced medication use [43··].

STOPPFrail version 2 was published in 2020, focusing on practicality and patient-centeredness. This version includes 25 medications and refined the definition of end of life as follows: (1) dependency in activities of daily living and/or severe chronic disease and/or terminal illness; (2) severe irreversible frailty; and (3) physician overseeing patient care would not be surprised if the patient died in the next 12 months [44··].

For cancer, OncPal was developed to aid deprescribing for patients transitioning from curative to palliative care [45·]. Based on a literature review and validated using an expert panel approach, there was excellent agreement (kappa = 0.83) between OncPal and an expert panel review ofdrugs. Among 61 patients with a life expectancy of < 6 months, the OncPal tool identified at least one inappropriate medication in 70% of patients.

### Additional Dimensions of Medication Appropriateness

Early studies of EOL medication appropriateness broadly divided medications into symptom management and chronic disease medications [5]. While intuitively appealing, a limitation of this approach is that there is no consensus definition for either category. Studies need to tailor and redefine these categories to meet the constraints of their unique data sources [46].

More recently, medication appropriateness screening tools have sought to extend beyond dichotomous determinations of appropriate vs inappropriate. Such binary measures largely fail to consider the relevant time point of medication use (i.e., drug initiation or continuation) and the level of potential appropriateness (i.e., adequate, questionable, inadequate). Morin et al [47] sought to improve medication appropriateness tools for older adults with a life expectancy (< 3 months). Using a Delphi consensus panel, they identified 14 drug classes deemed “often adequate,” 28 “questionable,” and 10 “often inadequate” medications for continuation. For medication initiation, the panel identified 10 “often adequate,” 23 “questionable,” and 23 “often inadequate” drugs. In subsequent work, the authors found that in the last 3 months of life, older adults were taking approximately 9 medications, and approximately one-third continued and 14% initiated at least one drug of questionable clinical benefit [48].

### Goal-Concordant Prescribing

The holy grail of care quality in end of life is goal-concordant care [49–51]. This concept is a nod to shared decision-making between clinicians and patients based on a partnership of equals [52] and focuses on the patients’ goals. This is a radical departure from outdated notions of compliance and adherence that reflect whether a drug is taken according to the clinicians’ orders [52]. At the end of life, goal-concordant prescribing would align medications prescribed with the patients’ goals for care. Thus, reductions in overall numbers of medications or complexity may not necessarily be desirable if the overall regimen is congruent with and able to achieve stated treatment goals. While conceptually desirable to include in deprescribing studies, how to measure this remains an ongoing challenge [53].

### Safety

Medication changes may result in positive or negative outcomes. Some studies use a capture of clinical events such as unplanned hospitalizations and emergency department visits to broadly measure adverse events [54, 55··] (Table 2). Inclusion of death in such measures, however, could be problematic because death is an expected in this terminally ill population. One approach used in a classic deprescribing trial was to compare occurrence of death within 60 days between discontinuation and continuation arms as evidence of safety for stopping the medication of interest [55··].

More specifically, of great interest in deprescribing is the occurrence of adverse drug withdrawal events (ADWEs), defined as a “clinically significant set of symptoms or signs caused by the removal of a drug” [56]. Also of concern are adverse drug events (ADE), defined as “any injury resulting from use of a drug” including noxious or unintended drug responses at normal doses [57]. A recent review of adjudication methods to identify ADWEs and ADEs is helpful to consider when thinking about measuring these outcomes [58··]. This adjudication review summarized approaches used by clinical experts, including the following: a probability scale questionnaire [59]; a Likert-scale based measure of clinical plausibility and implied causality [60]; expert clinical judgment [61]; case assessment combining chronological, clinical, and lab findings [62]; and case assessment using a causal relationships from de-challenge and re-challenge results [63], with a version including ADE avoidability [64]. Other approaches use the following: lab tests, causality, and drug administration timing to detect unknown and unexpected adverse events [65]; patient self-report [66]; or computerized ADE monitoring [67]. Notably, distinguishing between ADWEs and ADEs remains difficult. One strategy proposes to focus on adverse event identification, and then: “Once… identified, greater focus…on determining if it represents an ADE or ADWE by adapting an existing framework to meet the needs identified” [58].

While not recent, it is important to note that Hanlon et al. developed an approach that can be applied to medical record review or patient interviews [68]. In a seminal study of ADWEs [56], medical records were screened for potential ADWE signals, including symptoms, signs, abnormal laboratory results, or clinical events known to be associated with drug withdrawal in published literature. Automation of this, and all ADE/ADWE approaches, to electronic medical records or for clinical data such as the nursing home Minimum Data Set remains a significant challenge, with exploration of natural language processing offering a promising development [69].

### Patient-Reported Outcomes

Patient-reported outcomes capture aspects of end-of-life care that are important to patients and their families. Patient-reported outcomes, particularly relevant to end-of-life care, include quality of life and symptom control, both physical and psychological. There are several instruments to measure these outcomes. Fortunately, the Palliative Care Research Cooperative (PCRC) group (https://palliativecareresearch.org/) has developed a measurement tool library that contains 205 relevant and high quality instruments for palliative care research as of August 2020. For each instrument, the library contains a brief instrument description, the relevant disease setting, the target respondent, and administration procedures (number of items, requirement for a trained interviewer, recall period, and average time required). The library also provides an assessment of the instrument quality and key references.

The PCRC measurement tool library contains 26 measures that assess quality of life in end-of-life settings. Some instruments are specific to a disease or condition (e.g., Alzheimer’s disease, cancer), while others pertain to a given care setting (e.g., nursing home). These instruments range in time needed to complete the assessment from 5 min to 1 h. One example of a high quality–rated measure includes the McGill Quality of Life Questionnaire [70], which measures four subscales including physical symptoms, psychological symptoms, outlook on life, and meaningful existence, and a single item question about physical wellbeing.

The library also includes 12 measures, all rated as high quality, assessing a range of physical symptoms, 8 of which are general measures and not disease specific. One example of a physical symptom instrument is the Edmonton Symptom Assessment System [71]. This 10-item tool assesses physical and psychosocial symptom severity and can be applied in general palliative care settings. In addition, this instrument can be completed in approximately 5 min by a patient or their proxy, providing additional flexibility.

Finally, the library includes 9 measures that capture psychological symptoms, with all but one being general tools for use across disease settings. These tools broadly cover measurement of depression, agitation, mood, and communication ability. One example of a psychological symptom measurement tool is the Center for Epidemiologic Studies Depression Scale (CES-D) [72], which measures current depressive symptomatology in the past week. This 20-item instrument is one of the most widely used to measure depressive symptoms in research studies because of its excellent measurement properties, methodological quality, and consistency of results.

### Costs

Cost assessment following deprescribing is rarely performed; if it is, the costs assessed are heterogeneous. Statin discontinuation among patients at end of life reduced medication costs by $3.37 [55], using a national average retail price to calculate the avoided statin costs. A different study found significantly lower medication-related costs after deprescribing using the START/STOPP tool. Researchers counted the number of medications taken on the day of patient assessment and then summed medication costs using available price lists from the Israeli health ministry [73]. An intervention delivered to older adults with advanced cancer describes potential savings of $4200 by avoiding ADEs, plus reducing time to take medications histories or perform medication teaching [74]. The authors assumed that each discontinued medication resulted in the avoidance of a major or minor ADE. Prices were calculated using prices established by the health system in which the study took place.

### Caregiver Outcomes

Caregiver involvement is a unique aspect of end-of-life care that deserves attention in deprescribing studies, but these are largely underutilized in studies to date (Table 2). This can include measuring whether caregivers are open to deprescribing medications if a trusted clinician says stopping is possible [75]. Caregiver attitudes towards deprescribing can be assessed using the revised Patient Attitude Towards Deprescribing (rPATD) questionnaire, caregiver version [76].

### Medications Administration and Burden

Caregivers play an important role in medication management for patients, from obtaining and administering medications to monitoring for symptom relief and endorsing medication use. Most caregivers report spending significant time ordering or administrating medication for family members at end of life [77]. Caregivers have described polypharmacy burden and lack of clear direction from healthcare providers as significant sources of distress when caring for a dying loved one [78]. For older adults, the degree of caregiver involvement, hospice enrollment, decedent symptom-related distress, and financial burden are associated with higher caregiver burden [79]. The Family Caregiver Medication Administration Hassles Scale (FCMAHS) measures daily irritants associated with medication-related duties for caregivers; monitoring for an increase in irritations can facilitate interventions before significant caregiver distress occurs [80].

### Clinical Outcomes

Medication-related care may invoke anxiety among caregivers stemming from uncertainty around medication dosages and use of “as needed” medications and fear of overdosing patients, or committing medication-related errors that may shorten patient survival [81]. This may result in emergency department visits stemming from caregiver concerns about ability to manage symptoms and medications at home [82]. Factors contributing to caregiver distress include misunderstanding of pain medications, poor communication with clinicians, and inadequate knowledge of medications/assessment of pain symptoms [83]. Multiple tools are available to assess caregiver burden. The Caregiver Quality of Life Index [84] is available with multiple versions, including some specific to a particular condition such as cancer. The Ways of Coping Scale has been used to measure caregiver coping with patient symptoms and health [85]. Some caregivers report significant distress after observing patient symptoms at end of life, which can be assessed using the Stressful Caregiving Response to Experiences of Dying scale [86]. The Self-Perceived Pressure from Informal Care Scale and the Positive Experiences Scale have been used to measure caregiver burden and positive experiences stemming from providing care [87]. Other tools include the Zarit Burden Interview and the Center for Epidemiological Studies Depression Scale (CES-D) [88]. Following the loss of a patient, caregiver grief can be evaluated using the Inventory of Complicated Grief [89].

### Costs

Time costs are important, as caregivers provide on average 40–70 h of care a week to patients at the end of life [90, 91]. Caregiving-related costs can be calculated by surveying individuals, then assigning costs specific to reported activity. Higginson and colleagues surveyed bereaved caregivers using the Client Service Receipt Inventory to calculate hours spent caregiving. Then, the number of hours was multiplied by average hourly wages and cost of nursing care. In the last 3 months of life, average cost to informal caregivers in the USA was $32,468 (SD $28,578) [92]. An additional tool for cost measurement is the Ambulatory and Home Care Record [93]. Beyond hourly time and associated wages, caregiver diaries can measure out-of-pocket expenses and medication-related costs to caregivers and families. For example, caregivers can record all services used (e.g., caregiver respite not paid for by insurance) and expenses such as parking for doctor’s visits [94].

## Discussion

This narrative review summarizes existing approaches to the measurement of deprescribing and key outcome variables for studies examining deprescribing at the end of life. For each measure, there are several approaches to consider. The selection of appropriate measure follows from the method and study design, and design follows from the research question and the investigators’ vision for the study goals. Some studies implemented randomized clinical trials [43, 55], while others were observational pre-post studies [24, 40]. Of note is the small sample size of most of the available studies. As with all epidemiological studies, the final choices depend on the details and the perspective of the stakeholders who will utilize the study results.

Since patients are central to all deprescribing studies, and the goal of deprescribing is to reduce medication burden and improve (or maintain) quality of life [1], we have summarized approaches to capturing these measures as they have been operationalized to date. Somewhat unique to EOL research is the perspective of both the patient and the caregiver. We have summarized measures including burden of medication administration and caregiver quality of life, which rely on primary data collection. While costs are important to patients and caregivers, detailed costing methods are beyond the scope of this paper. Issues such as the distinction between out-of-pocket costs vs total costs and costs versus charges are important to consider in this domain.

We suggest addressing the following issues before the application of polypharmacy as an outcome variable for deprescribing interventions in the end-of-life population.

First, identify the goal of the study and what data are available. Patient-level prescription data from medication charts can provide an accurate “point prevalence” of patients’ medication use. With individualized medication history to define polypharmacy, providers could compare the drug list with medical history to initiate and follow the deprescribing process. However, such studies are limited to the end-of-life population in well-defined settings leading to limited external validity, making it difficult to validate the outcome for the deprescribing intervention and to generalize to a larger population. Alternatively, studies using registry or administrative databases can be generalized to a broader population but cannot capture the effect on individualized patient-level-deprescribing processes. Such studies can assess the medications that the patients were prescribed or dispensed, but may not reflect what the patient actually took.

Second, identify the definition of polypharmacy. Three questions should be asked. First, what medications should be included? Although the types of medications counted towards polypharmacy are generally “chronic” medications, they may vary with regard to the specific health conditions of the end-of-life population in the study. Second, how long should medications use be measured? This depends on medication type and patients’ conditions. If studies included drugs for symptom management or preventive medications in the total polypharmacy count, does the assessment time length for these drugs differ from the chronic medications that were also included? Lastly, given the baseline medication use of the study population, the common threshold of 5 or 10 medications might not be an optimal cut-off. If many medications are deprescribing targets for an end-of-life population of interest, a more conservative threshold of greater number of medications may be necessary.

For both deprescribing and outcome measures, there are differences in definitions based on whether primary data (e.g., survey) or secondary data (e.g., administrative claims or medical records) are used for assessment. Clinical outcomes such as mortality or healthcare utilization can be garnered from claims and medical records. Patient-reported outcomes such as symptom burden and quality of life are mostly reliant on primary data collection, but investigators are starting to explore methods for using secondary data sources such as the Minimum Data Set for nursing home residents. At the edge of existing methods are theoretically important outcome measures like goal-concordant prescribing that need further study for definition and operationalization from data sources. Data captured by a given resource often limits the range of variables available for inclusion. For example, there may be subtle but important differences between drug prescribing orders from medical records, drug dispensing from pharmacy records, and drug claims from administrative records.

Final selection of measures should follow from basic epidemiologic design, which requires that the investigator weigh measures based on balance of validity, reliability, feasibility, and acceptability. Feasibility and acceptability are particularly challenging in end-of-life research as primary data collection from patients who are seriously ill or dying, and their caregivers who are bereaved, is considered ethically challenging. Given these complex trade-offs, the use of a stakeholder panels can help inform study design decisions.

### Limitations

Our review is limited to patient and caregiver perspectives. We did not include measures reflective of other stakeholders, including clinicians and payers [95]. Specifically, we did not review healthcare utilization measures. We focus largely on older adults in this review, which represents the available literature. We do not address the issue of how to define end-of-life, which has no widely accepted criteria to operationalize. This is an important issue for identifying the population under study and enumerating an appropriate denominator [18]. Such definitions are important to note because they affect the magnitude of the prevalence of deprescribing and medication appropriateness.

## Conclusion

Deprescribing studies at EOL should consider medication types, amounts, and appropriateness in the context of patient goals of care. Additional data are needed on deprescribing at EOL in populations other than older adults. Future research should evaluate the association between deprescribing and patient quality of life, caregiver burden, and out-of-pocket medication-related costs to patients and families.

## Supplementary Material

Supplemental data

## Figures and Tables

**Table 1:** Deprescribing measurement in end-of-life studies

Domain	Proposed measure	Outcome Measure Operationalization in Selected Studies	Example Study with Data Source(s)	Research Gaps/Notes
**Deprescribing**				
**Discontinuation**	**Days Gap Windows**	**Administrative data**		Defining discontinuation from administrative data needs to address how to handle restarts of either the same medication or medication within the same class for a designated window period. Defining discontinuation using medical records, e.g. in intervention studies, is based on the randomization or the recorded order for discontinuation and not subject to inferences needed for administrative data.
		Operationalization: 7-day Rap with 30-day follow-up period	Vu et al. 2021 (16) Data: VA bar code medication administration records and Minimum Data Set linked to Medicare claims	
		Operationalization: 14-day gap with 90-day follow-up period	Thorpe et al. 2020 (17) Data: VA bar code medication administration records and Minimum Data Set linked to Medicare claims	
		Operationalization: 30-day gap with 30-day follow-up period	Mack et al. 2020 (18) Data: Minimum Data Set 3.0 merged to Medicare administrative claims	
**Deintensification**	**Gradual Dose Reduction** **Drug class-specific strategies proposed** (22) Central nervous system (CNS) drugs - medication intensity considered dividing a patien’s daily dose by defined daily dosage (DDD), or the minimum effective adult or geriatric daily dosage. Anti hypertensive drug - intensity consider either DDD or maximum recommended daily dosage in the denominator.	**Administrative data**		Machine learning using methods such as national language processing could theoretically detect explicit notation of deliberate deintensification/dose reductions in the medical records
		Operationalization: Dose reduction in total dailv dose from medication administration records for at least 7 days	Vu et al. 2021 (16) Data: VA bar code medication administration records and Minimum Data Set linked to Medicare claims	

**Table 2:** Outcome measures in end-of-life deprescribing studies

Domain	Proposed measure	Outcome Measure Operationalization In Selected Studies	Example Study with Data Source(s)	Research Gaps/Notes
**Patient Focused Measures**				
**Medication-related Outcomes**													
**Number of Medications**													
**Polypharmacy**	Conceptually defined as if the total number of medicationstaken by a patient In a given period of time surpassed the pre-specified threshold. Thresholds commonly used are (1) 5 or more medications (polypharmacy) and (2) 10 or more medications (excessive polypharmacy).	**Chart review**		Limited availability of drugs included in administrative dataset (Minimum Data Set) Inconsistency in the measurement time period Inconsistency in how the medications were classified (e.g. ATC) Inconsistency in the types of the medications would be counted towards the measure of polypharmacy. (e.g. chronic prescriptions only or all including *prn*) Inconsistency in the study populations (definitions of end-of -life; decedents vs cohort approaching death)
		**Operationalization:** Polypharmacy based on total number of chronic drugs taken by resident for >= 6 months	**Molist-Brunet 2020 (96)** Data: Collected pharmacological data reviewed through a systematic 4-stage pharmacotherapy process by a multi-disciplinary team (geriatrician, nurse, clinical pharmacist)	
		**Operationalization:** Polypharmacy was defined as the use of five or more prescribed chronic medications with *systemic effects*, and excessive polypharmacy as the use of ten or more.	**Paque 2019a (27)** Data: Medication use was based on a copy of the residen’s full medication chart and was evaluated two times: (t2) at the time of data collection and (t1) retrospectively 3 to 6 months before. Medications were classified using the Anatomical Therapeutic Chemical (ATC) classification	
		**Operationalization:** Polypharmacy was defined as the use of five or more prescribed chronic medications, and extreme polypharmacy as the use of ten or more.	**Paque 2019b (26)** Data: Medication use based on full medication charts. Classified using ATC	
		**Operationalization:** Polypharmacy was defined as 5 or more medications in the last year of life	**Ho 2020 (25)** Data: Case record review	
		**Operationalization:** Polypharmacy defined as 5 or more different drugs per day. We determined the number of each prescribed medication at four predefined time points: 9 (=day–9), 6 (=day–6), 3 (=day–3) days before death and the day of death (=day 0).	**Kierner 2016 (23)** Data: Medical charts	
		**Linked registries**		
		**Operationalization:** Polypharmacy based on monthly exposure to 10 or more prescription drugs (authors opted for the more conservative cut-off of 10 In light of the chronic diseases and symptoms towards the end of life), that is, distinct substances according to the fifth level of the ATC classification system	**Morin et al 2017** (**26)** **Data: Nationwide study** of linked registries: death certificate data, Swedish prescribed drug register, social services register, national patient register, Swedish education register	
		**Operationalization:** Polypharmacy based on monthly use of ten or more different prescription drugs. ATC classification.	Grande 2017 (29) Data: Death certificate data linked at the individual level with the Swedish Prescribed Drug Register (ATC classification), the National Patient Register, and the Social Services Register.	
		Administrative dataset		
		**Operationalization:** polypharmacy defined as 9+ medication based on the last MDS 2.0 assessment before death	Hoben 2016 (30) Data: Resident Assessment Instrument-Minimum Data Set 2.0 (2007–2012)	
Medication Regimen				
**Regimen Complexity**	**Medication Regimen Complexity Index (32)** 65-item quantitative and validated instrument that weights the average number of medications, dosage, frequency, and administration	**Chart review**		Users manually enter the dosing and route form in the “automated MRCI” version. (37) Development of a fully automated version from electronic medical record or administrative data yet to be published.
		Operationalization: data entry tool available at https://pharmacy.cuanschutz.edu/research/MRCTool for Microsoft Access database	**Walter 2020 (36)** Data: Medical record review	
**Medication Appropriateness**				
**Explicit Criteria**	**STOPP Frail V1 (41) & STOPP Frail V2 (44)** STOPPFrail is a list of potentially inappropriate prescribing indicators designed to assist physicians with stopping such medications in older patients (≥65 years) who meet ALL of the criteria: (1) End-stage irreversible pathology; (2) Poor one year survival prognosis; (3) Severe functional impairment or severe cognitive Impairment or both; (4) Symptom control is the priority rather than prevention of disease progression	**Intervention Trial**		Explicit criteria can overlook importance of implicit (judgement-based) identification strategies which can be difficult to operationalize in administrative data Limited availability of drugs included in some administrative datasets (e.g. Minimum Data Set) STOPPFrail be difficult to match in an claims data not supplemented with clinical data to the intended audience of patients who meet the criteria of irreversible pathology, poor 1-year survival, functional impairment/cognitive impairment and goal of symptom control
		Outcome measures: Primary - polypharmacy (five or more drugs) at 3 months and PIMs determined by medication appropriateness index (MAI). Secondary - unscheduled hospital presentations, falls, quality of life, monthly medication costs, and mortality.	**Curtin 2020 (43)** Data: Case report form capture of hospital medication record reviews	
		Chart Review		
		Outcome measures: medication consumption was determined by examining hospital Medication Administration Records. Potentially inappropriate medications were defined using STOPPFrail deprescribing criteria.	**Curtin 2018** (97, 98) Data: Medication Administration Records	
	**OncPal Deprescribing Guideline (OncPal) (45)** The OncPal Deprescribing Guideline was created by investigating the current literature for the de-escalation of medications by systematically reviewing each medication class according to the European Pharmaceutical Market Research Association anatomical classification list	**Observational**		Highly sensitive for deprescribing in patients with complexities such as having chronic diseases with different associated co-morbidities or multimorbidity. Does not include some medication classes that might have been identified as PIM e.g., antidepressants.
		Measure: mean number of medications; Potentially inappropriate medication defined by OncPal	**Wenedy 2019 (99)** Data: Hospice records	
	**Preventive and Symptomatic**	**Observational Study**		Definitions of symptomatic and preventive drugs are not universal and have to be defined for each study.
		Operationalization: Unnecessary preventive drugs and symptomatic drugs defined by clinician	**Pasina 2019 (46)** Data: Hospice records	
**Implicit Criteria**	**Unnecessary Drug Use Measure** (3-item Medication Appropriateness Index [MAI])	**Retrospective observational**		Requires manual review and adjudication.
		Operationalization: number of unnecessary medications by 3 item MAI based on expert review	**Suhrie 2009 (40)** Data: VA medical records	
**Safety**				
**Adverse Events**	**Adverse Drug Withdrawal Events (ADWE) & Adverse Drug Events (ADE)**	Intervention trial		There is no standardized approach to defining ADWE and ADE in patients at end of life.
	Approaches to identification and classification typically include adjudication based on clinician review of data abstractions (58) Potential adverse events can also be prespecified defined by investigators (54, 55)	Operationalization: Prespecified adverse events monitored at each assessment included hospital admissions, emergency department visits, new cardiovascular events, invasive procedures for cardiac events, venous thromboembolism, and pneumonia. Ad hoc adverse events were documented and monitored by site Investigators.	**Kutner 2015 (55)** Data: Medical record abstractions in intervention trial	
		**Observational study**		
		Operationalization: Potential ADE/ADWE as survival, falls, fractures, hospital admissions, cognitive, physical, and bowel function, quality of life, and sleep (secondary outcomes)	**Potter 2016 (54)** Data: Medical records	
**Patient Outcomes**				
**Patient-Reported Outcomes**	**Quality of Life**	Intervention trial		Relies on primary data collection Best used with trial populations Application in secondary data may be possible using sources such as the Minimum Data Set in nursing home residents, but is unexplored.
		Operationalization: McGill Quality of Ufe (70). A single-item overall QOL score and subscales (physical symptom, psychological symptom, existential well-being, and support)	**Kutner 2015 (55)** Data: Primary data collection from intervention study	
	**Symptoms**	Interventfon trial		
		**Operationalization:** Edmonton Symptom Assessment Scale (71). The 9 standard items on the scale (pain, fatigue, nausea, depression, anxiousness, drowsiness, appetite, well-being, and breathing)	**Kutner 2015 (55)** Data: Primary data collection from intervention study	
**Costs**				
**Savings**	**Potential Savings from Avoiding Adverse Events**	Operationalization: Potential savings by avoiding medication-related adverse events, plus reducing time needed to take medications histories and perform medication teaching because the patients received fewer medications following deprescribing.	Not yet used in EOL deprescribing studies.	
**Expenditures**	**Medication Costs**	**Intervention trial**		
		Operationalization: Calculated the 28-day cost of participants’ prescription drugs using a pharmaceutical wholesaler price list. For each specific medication dose and formulation, the lowest cost option was chosen.	**Curtin 2020 (43)** Data: Medical record	
	**Patient-specific monthly cost**	**Intervention trial**		
		Operationalization: Estimated cost savings resulting from statin discontinuation by first converting monthly to daily costs and then tracking the avoided costs from the time each patient was randomized until death or censorship.	**Kutner 2015 (55)** Data: National average retail price compiled by *Consumer Reports*	
**Caregiver Focused Measures**				
**Attitudes**	**Satisfaction with Care**	**Intervention trial**		
		Operationalization: Satisfaction with health care, assessed by likelihood to recommend current health care on a Likert scale	**Kutner 2015 (55)** Data: Intervention primary data collection	
	**Deprescribing**	Operationalization: Patient Attitude Towards Deprescribing (rPATD), caregiver version. (76)	Not yet reported in EOL deprescribing studies	
**Administration and Burden**	**Medication Administration Hassle**	**Intervention trial**		
		Operationalization: The Family Caregiver Medication Administration Hassles Scale. (80) Measures daily irritants associated with medication-related duties for caregivers; monitoring for an Increase In irritations can facilitate interventions before significant caregiver distress occurs.	**Tjia 2020** (Clinicaltrials.gov NCT03972163) Data: Intervention primary data collection	
	**Time**	Operationalization: Self-reported time ordering medicine or performing medication for family members at the end of their life. (77)	Not yet reported in EOL deprescribing studies	
**Clinical Outcomes**	**Quality of Life**	Operationalization: The Caregiver Quality of Life Index. (84) Self-Perceived Pressure from Informal Care Scale and the Positive Experiences Scale (87)	Not yet reported in EOL deprescribing studies	
	**Coping**	Operationalization: The Ways of Coping Scale (WoCS). (85) Measures how people cope with stressful encounters, and has been used to measure caregiver coping with patient symptoms and health	Not yet reported in EOL deprescribing studies	
	**Stress with Death**	Operationalization: Stressful Caregiving Response to Experiences of Dying (SCARED) scale. (86)	Not yet reported in EOL deprescribing studies	
**Costs**	**Caregiver Hours**	Operationalization: Client Service Receipt Inventory to calculate hours spent caregiving, multiplying by average hourly wages and cost of nursing care. (92)	Not yet reported in EOL deprescribing studies	
		Operationalization: An additional tool for cost measurement is the Ambulatory and Home Care Record (93)	Not yet reported in EOL deprescribing studies	
	**Out-of-Pocket Costs**	Operationalization: Caregiver diaries can be used to measure out-of-pocket expenses and medication-related costs to caregivers and families. Caregivers can record all services used (e.g. caregiver respite not paid for by insurance or hospice) and expenses such as gas and parking for doctor’s visits over a 2-week period. (94)	Not yet reported in EOL deprescribing studies	
