# Supplementary material for: Methodological Challenges for Epidemiologic Studies of Deprescribing at the End of Life
Source: Curr Epidemiol Rep. Author manuscript; Available in PMC 2021 Oct 28. (PMC8553236; doi:10.1007/s40471-021-00264-7)
Supplement: Supplemental data [file NIHMS1700960-supplement-Supplemental_data.pdf]

**Supplemental Table. Examples of common drugs deprescribed and recommended deprescribing regimens**		
**Common Class of Drug Deprescribed**	**Deprescribing Recommendation**	**References**
Antihypertensives	Abrupt discontinuation may be safe for most populations with well controlled hypertension. The exception is for clonidine and beta blockers, of which tapering is required for safe withdrawal to avoid rebound effects.<br><br>The specific time period of tapering varies by each drug, its pharmacokinetic properties, and the population.	Steinman M, Reeve E, Schmader K, Givens J. Deprescribing. UpToDate. 2020; Available from: https://www.uptodate.com/contents/deprescribing?search=deprescribing&sectionRank=1&usage_type=default&anchor=H2935778044&source=machineLearning&selectedTitle=1~150&display_rank=1#H3123243208<br><br>Van Der Wardt V, Harrison JK, Welsh T, Conroy S, Gladman J. Withdrawal of antihypertensive medication: A systematic review. J. Hypertens. 2017; 35(9):1742–9.
Proton Pump Inhibitors	Based on evidence, it appears that proton pump inhibitors are most effectively deprescribed with tapering compared to abrupt discontinuation.<br><br>Recommended tapering regimens will depend on each specific prescription drug and the targeted population. One deprescribing approach to these medications that has been suggested includes tapering the dose by 50% for two to four weeks and then complete withdrawal.	Haastrup P, Paulsen MS, Begtrup LM, et al. Strategies for discontinuation of proton pump inhibitors: a systematic review. Fam Pract 2014; 31:625.<br><br>Kim J, Blackett JW, Jodorkovsky D. Strategies for Effective Discontinuation of Proton Pump Inhibitors. Curr Gastroenterol Rep 2018; 20:27.
Diabetes Medications	These medications could be targeted for deprescribing when less medications are necessary to achieve glycemic goals or risks outweigh benefits, especially for older adults. Deprescribing these medications are often approached with deintensification, gradual dose reduction, or replacement therapies. In certain contexts, when hyperglycemia is not a serious risk, complete discontinuation may be safe. However, limited evidence exists to recommend more specific strategies for deprescribing these medications in various populations.	American Diabetes Association. 12. Older Adults: Standards of Medical Care in Diabetes-2019. Diabetes Care 2019; 42:S139.<br><br>Seidu S, Kunutsor SK, Topsever P, Hambling CE, Cos FX, Khunti K. Deintensification in older patients with type 2 diabetes: A systematic review of approaches, rates and outcomes. Diabetes, Obes Metab. 2019;21(7):1668-1679.
Statins	Statins can be discontinued completely in appropriate patient populations without tapering, particularly in those with life-limiting illness.	Kutner JS, Blatchford PJ, Taylor DH, Jr., Ritchie CS, Bull JH, Fairclough DL, et al. Safety and benefit of discontinuing statin therapy in the setting of advanced, life-limiting illness: a randomized clinical trial. JAMA Intern Med. 2015;175(5):691-700.
Psychoactive Medications	Cognitive altering medications include memantine or cholinesterase inhibitors, benzodiazepines, benzodiazepine receptor agonists etc). In general because of the potential dependence of these medications both psychologically and physiologically, to safely deprescribe these medications tapering is recommended.	Steinman M, Reeve E, Schmader K, Givens J. Deprescribing. UpToDate. 2020; Available from: https://www.uptodate.com/contents/deprescribing?search=deprescribing&sectionRank=1&usage_type=default&anchor=H2935778044&source=machineLearning&selectedTitle=1~150&display_rank=1#H3123243208

	For cholinesterase inhibitors and memantine, a stepwise dosing reduction is recommended. For example one recommendation is halving the dose every four weeks until complete withdrawal.<br><br>For benzodiazepines a stepwise tapering regimen is also recommended with ideally 25% dose reduction biweekly and 12.5% reduction before complete withdrawal.	Reeve E, Farrell B, Thompson W, et al. Deprescribing cholinesterase inhibitors and memantine in dementia: guideline summary. Med J Aust 2019; 210:174.<br><br>Pottie K, Thompson W, Davies S, et al. Deprescribing benzodiazepine receptor agonists: Evidence-based clinical practice guideline. Can Fam Physician 2018; 64:339.<br><br>Benzodiazepine and Z-Drug (BZRA) deprescribing algorithm. 2019. https://deprescribing.org/wp-content/uploads/2019/03/deprescribing_algorithms2019_BZRA_vf-locked.pdf
